# Towards functional spin-echo BOLD line-scanning in humans at 7T

**DOI:** 10.1007/s10334-022-01059-7

**Published:** 2023-01-10

**Authors:** Luisa Raimondo, Jurjen Heij, Tomas Knapen, Serge O. Dumoulin, Wietske van der Zwaag, Jeroen C. W. Siero

**Affiliations:** 1 Spinoza Centre for Neuroimaging, Meibergdreef 75, 1105 BK Amsterdam, The Netherlands; 2 Computational Cognitive Neuroscience and Neuroimaging, Netherlands Institute for Neuroscience, Meibergdreef 47, 1105 BA Amsterdam, The Netherlands; 3 Experimental and Applied Psychology, VU University, De Boelelaan 1105, 1081 HV Amsterdam, The Netherlands; 4 Experimental Psychology, Utrecht University, PO Box 80125, 3508 TC Utrecht, The Netherlands; 5 Radiology, University Medical Centre Utrecht, Heidelberglaan 100, 3584 CX Utrecht, The Netherlands

**Keywords:** Line-scanning, High spatiotemporal resolution, fMRI, 7T, Spin-echo

## Abstract

**Objective:**

Neurons cluster into sub-millimeter spatial structures and neural activity occurs at millisecond resolutions; hence, ultimately, high spatial and high temporal resolutions are required for functional MRI. In this work, we implemented a spin-echo line-scanning (SELINE) sequence to use in high spatial and temporal resolution fMRI.

**Materials and methods:**

A line is formed by simply rotating the spin-echo refocusing gradient to a plane perpendicular to the excited slice and by removing the phase-encoding gradient. This technique promises a combination of high spatial and temporal resolution (250 μm, 500 ms) and microvascular specificity of functional responses. We compared SELINE data to a corresponding gradient-echo version (GELINE).

**Results:**

We demonstrate that SELINE showed much-improved line selection (i.e. a sharper line profile) compared to GELINE, albeit at the cost of a significant drop in functional sensitivity.

**Discussion:**

This low functional sensitivity needs to be addressed before SELINE can be applied for neuroscientific purposes.

**Supplementary Information:**

The online version contains supplementary material available at 10.1007/s10334-022-01059-7.

## Introduction

Functional magnetic resonance imaging (fMRI) is a powerful tool in neuroscience to detect brain activity, particularly based on the blood oxygenation level-dependent (BOLD) signal [1]. Neurons cluster into sub-millimeter columnar and laminar structures and neural activity occurs at millisecond resolution; hence, when investigating brain activation differences across layers, high spatial and high temporal resolution is required. The recently described gradient-echo line-scanning (GELINE) sequence achieved very high resolution in humans [2, 3] across cortical depth (250 μm) and time (~ 200 ms), by sacrificing volume coverage and resolution along the cortical surface. This method is based on very early MRI experiments [4, 5] and was more recently implemented in rodents [6] and for relaxometry and diffusion MRI in humans [7]. Gradient-echo (GE) BOLD is highly sensitive to changes in the local T2* relaxation time and is the most commonly used contrast in fMRI experiments. However, it suffers from non-specific signal contributions from large veins [8–10]. This is even more problematic when high spatial resolution is involved since confounds caused by signals from non-capillary vessels impact the localizational fidelity of the GE BOLD fMRI signal [11, 12]. Hence, more specific functional imaging techniques have recently gained much attention [13–17]. Spin-echo (SE) functional responses are expected to be much better localized to the site of neuronal activation, because of the strong micro-vascular weighting which can be achieved with SE for field strengths larger than 3T [10, 11, 18–20]. In fact, this technique offers a better localization of the signal coming from the capillaries, particularly at ultra-high magnetic field strength (7T and above) and presents the advantage of furnishing an optimal sensitivity with a single echo readout, due to the little variation in T2 of gray matter (GM) through the brain (unlike the considerable variation of T2*, in case of GE BOLD) [20]. Although spin-echo imaging is long established for fMRI [21–23], the technique continues to be developed to improve sensitivity and acquisition efficiency [16, 24–28].

The high spatiotemporal resolution reached with line-scanning, when combined with a functional contrast more specific to the microvasculature than GE BOLD, would allow us to isolate microvessel responses and to characterize the distribution of blood flow and laminar fMRI profiles across cortical depth with higher fidelity. Moreover, spin-echo line-scanning (SELINE) offers beam excitation without the need for the outer-volume suppression (OVS) pulses, which are necessary in the case of GELINE and lead to imperfect RF saturation performance, hence poor line boundary definition [4]. SELINE capitalizes on a simple rotation of the plane for the refocusing pulse to a perpendicular plane. This intrinsic characteristic of SELINE allows us to minimise out-of-line signal contributions. Theoretically, it also results in lower specific absorption rate (SAR) limits, because of the absence of OVS pulses, even if SAR restrictions arise due to the presence of a refocusing pulse.

Besides the numerous advantages that SE would give to line-scanning, it also presents intrinsic problems when combining the formation of a SE with high-resolution fMRI. First, the need for a relatively long TE (~ 50–55 ms) at 7 T to match the T2 of cortical gray matter [29]. This makes it more difficult to keep a short TR for fMRI studies; this aspect is even more important for line-scanning fMRI, which is characterized by a high temporal resolution. Second, the TR is limited by the need to have at least one refocusing radiofrequency pulse in the sequence, which can also reach the power limits (SAR) with very short TRs. Third, SE has an intrinsically lower BOLD sensitivity and tSNR compared to GE (approximately half the BOLD percentage signal change and tSNR for SE compared to GE) [11, 30, 31]. Finally, the signal-to-noise ratio (SNR) of SE is lower than GE, making small voxels more difficult and increasing the required measurement times.

Spin echo lines have been implemented for diffusion-weighted MRI, to show the laminar architecture of the primary somatosensory cortex and primary motor cortex at 250–500 μm spatial resolution [7].

Here, we present our implementation of SELINE for BOLD fMRI in humans at 7T. We compared the performance of SELINE with a GELINE acquisition in terms of temporal signal-to-noise ratio (tSNR), line specificity and BOLD sensitivity. We also guide the reader through specific technical issues we ran into during the implementation of the sequence.

## Methods

We scanned 5 healthy participants at a 7T MRI system (Philips, Netherlands) equipped with a 2 channel transmit, 32 channels receive head coil (Nova Medical, USA) and 1 participant with an 8 channel transmit, 32 channels receive head coil (Nova Medical, USA). In addition, multiple pilots were conducted to optimize the parameters used here (Table 1). For pilot studies, we used a sphere phantom or healthy participants.

**Table 1:** Sequence parameters for pilot SELINE acquisition

N transmit channels	TR [ms]	TE [ms]	FA [deg]	BW [Hz/pixel]	fat suppression
2	500	50	146	28.89	SPAIR
8	300	40	148	28.95	SPIR
8	200	50	154	28.95	SPIR
8	190	43	154	28.95	SPIR
8	190	40	154	28.95	SPIR
8	355	40	146	28.95	SPAIR
8	500	50	140	28.95	SPAIR

*FA* flip angle, *BW* bandwidth

All participants provided written informed consent before participating, and the study was approved by the local ethical committee.

**Fig. 1:**
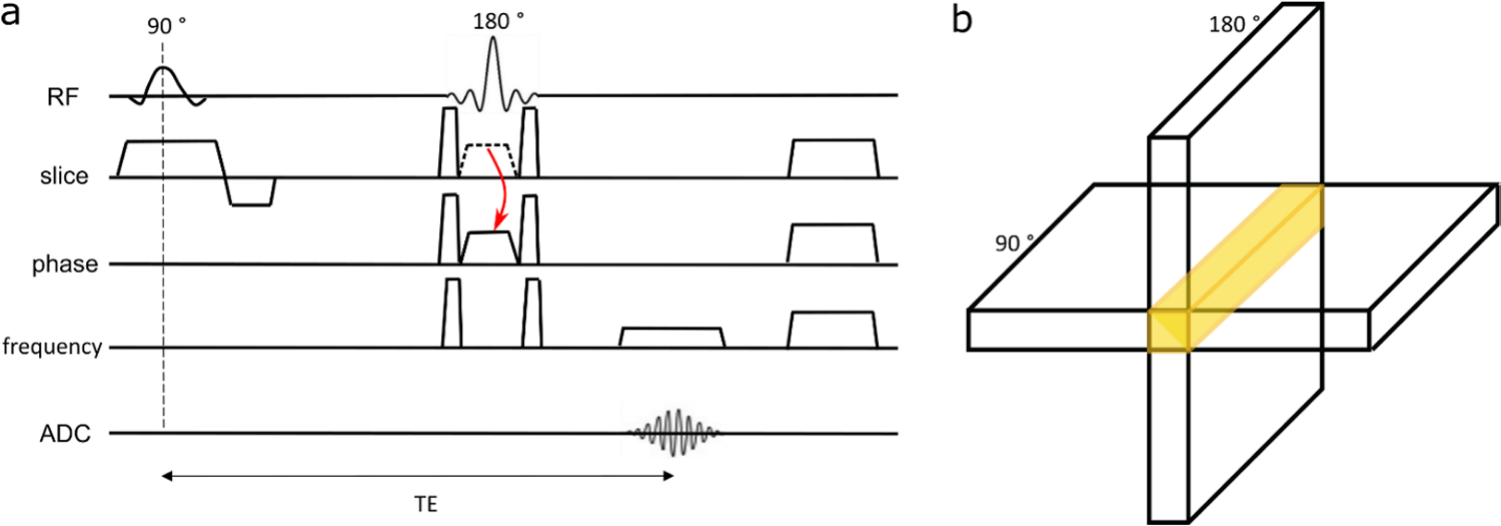
**a** SELINE sequence. Note the absence of phase-encoding gradients and the two different orientations of the 90° and 180° gradients to excite and refocus perpendicular planes. The gradient was moved from the slice-selection direction to the phase-encoding direction, as indicated by the red arrow. Pairs of crusher gradients were added around the refocusing gradient in every direction to avoid free induction decay artefacts, while spoilers were introduced at the end of the sequence to eliminate residual transverse magnetization. **b** Excitation and refocusing of 2 perpendicular planes, leading to a signal coming from a beam, indicated in yellow

We modified a 2D spin-echo sequence for the SELINE data acquisition (Fig. 1a). The phase-encoding in the direction perpendicular to the line was turned off. Other parameters: line resolution 250 μm, TR 500 ms, TE 50 ms, flip angle 146°, array size 720, line thickness 2.5 mm, in-plane line width 5 mm, fat suppression using the vendor implementration of SPectral Attenuated Inversion Recovery (SPAIR), adjusting the frequency offset to 250 Hz and bandwidth (BW) to 1000 Hz. Different 180° refocusing pulse shapes were tested, and the one leading to the best results in terms of beam selection was a symmetric sinc pulse named ‘echo2’ in the vendor software. It has a maximum B_1_ of 18 μT and a bandwidth-time product of 4.4. Its slice-selection gradient was moved to the phase-encoding direction to refocus only a single beam of the excited slice (Fig. 1b). Note that with “beam selection” we mean the formation of the line as a result of the intersection between the excited plane and the refocusing of the perpendicular plane.

Pairs of crusher gradients (strength = 25mT/m, duration = 1.9 ms) were added around the refocusing gradient in every direction to avoid free induction decay (FID) artefacts while spoilers (strength = 3.3mT/m, duration = 21.6 ms) were introduced at the end of the sequence to eliminate residual transverse magnetization. The readout was performed with a gradient duration of 19.5 ms and a strength of 2.6 mT/m.

GELINE data acquisition was based on the method described previously [2]. Briefly, line-scanning data using a modified 2D gradient-echo (GE) sequence, where the phase-encoding gradients were turned off. Before slice excitation, the signal outside the line of interest was suppressed through two slab-selective spatial radiofrequency (RF) saturation pulses for outer volume suppression (OVS). The spatial saturation pulses had a pulse duration of 7.16 ms, a pulse flip angle of 97°, RF amplitudes of 4.85mT and 4.67 mT, respectively, and selection gradients with 0.27 mT/m gradient strength and duration of 7.76 ms. Fat suppression was applied before the OVS using the vendor implementation of spectral presaturation with inversion recovery (SPIR), adjusting the frequency offset to 250 Hz and bandwidth to 1000 Hz. The other parameters were: line resolution of 250 mm, array size 720 points along the line, and line thickness in the ‘slice’ direction 2.5 mm. The nominal in-plane line width was 4 mm, flip angle of 16° and TE 22 ms. This TE was used to achieve an optimal GE BOLD contrast. We used a slightly lower TE than the T2* of gray matter at 7T [32] to compensate for T2*-shortening by B_0_ inhomogeneities and to increase the SNR. The readout gradient duration of 22.28 ms and the strength of 4.26 mT/m, resulting in a readout bandwidth of 45.4 Hz/pixel. A TR of 500 ms, however, was used here to match that of the SELINE acquisition in terms of temporal resolution and degrees of freedom in the GLM for assessing significant task activation.

Regarding the line position, since GELINE and SELINE are scanned in the same session, it is possible to use exactly the same geometry for both acquisitions. In fact, the slice parameters were copied from one acquisition to the other, while the lines end up in the middle of the slice in both cases (either through the rotation of planes or through the placement of saturation slabs).

SELINE and GELINE data were reconstructed offline using Matlab (Mathworks Inc, USA) and MRecon (Gyrotools, CH). Multi-channel line data were combined with a weighted sum of squares (SoS), based on tSNR and coil sensitivity maps (csm) per channel:

1 $$S\left(x\right)=\frac{{\sum }_{i}^{{N}_{c}}{w}_{i}\left(x\right)*{S}_{i}(x)}{\sqrt{{\sum }_{i}^{{N}_{c}}{\left|{w}_{i}\left(x\right)\right|}^{2}}},$$

where *S* is the MRI signal, *Nc* is the number of channels of the receive coil (*Nc* = 32), and ${w}_{i}\left(x\right)=conj(csm)* tSNR\left(x\right) per\, coil$ as the weighting factor. Details for the reconstruction are reported in Raimondo et al. [2]. In addition to the previously described pipeline, we introduced a NORDIC-based denoising step to remove thermal noise prior to the coil combination step [33, 34].

For both acquisitions, the line was positioned perpendicular to the visual cortex as much as possible, considering the restrictions to the geometry, allowing only coronal acquisitions with the line positioned in the center of the slice and 45° angulation away from a coronal plane. The occipital lobe was identified from a low-resolution whole brain scan, and a coronal slice covering a portion of the visual cortex was scanned. From that slice, a portion of gray matter was selected and the left-to-right oriented line was placed to cover that region. Sometimes more than one slice was scanned, to make sure that the line intersected a suitably positioned portion of gray matter. The whole planning procedure took around 5 min. By design, the line was centered in the middle of the slice for both SELINE and GELINE, hence, in the absence of gross subject motion, further registration of the line to the slice was unnecessary.

We acquired one run of functional data with each protocol, using a block design visual task consisting of an 8 Hz flickering checkerboard presented for 10 s on/off. Runs lasted 6 min and 20 s, starting with a 10 s baseline period. For one subject, 2 GELINE runs and 4 SELINE runs were acquired to further increase the functional SNR, when averaging more runs. Note that we scanned a highly experienced participant when we acquired more runs to make sure that motion was not degrading the data quality; for this reason we did not perform any kind of registration between lines. Functional data were analyzed using a general linear model (GLM) approach, and t statistical values (t-stats) were evaluated to identify active voxels.

We also ran an independent component analysis (ICA) on both GELINE and SELINE data. This validation is useful when there is no certainty that the temporal autocorrelation is handled properly [35].

We also calculated the tSNR across the line, as well as in an 11 voxels ROI containing gray matter, through

2 $$tSNR=\frac{\overline{S(t)}}{\sigma (S\left(t\right))},$$

where $\overline{S(t)}$ is the mean signal over the whole time course, and $\sigma (S\left(t\right))$ is the standard deviation of the signal across time for the whole time course. For each subject, we acquired a Line Signal Distribution (LSD) image with the same parameters used for the SELINE acquisition but without the removal of the phase encoding gradient. The LSD image describes the imaged line, hence the line-scanning sequence without the removal of the phase-encoding gradients. Additionally, for one subject, we acquired matched 2D gradient-echo (GE) and SE EPI fMRI at a lower spatiotemporal resolution (1.5 mm isotropic, TR = 2.5 s, but with the same TE, and ‘coverage’ as the line images) to compare functional runs in terms of t-stats of single slices with line scanning fMRI. In addition, we acquired LSD images (with the same parameters) with the same visual task to investigate the effect of line selection on functional sensitivity in both acquisitions.

### Design considerations

Here, we summarize minor technical constraints we encountered during the implementation of the sequence:

- Spoiler gradients were added at the end of the TR loop on all 3 gradient axes to avoid phase artifacts in the center of the slice image (see Fig. S1a and b). Those spoiler gradients of 3.3 mT/m strength and 21.6 ms duration completely eliminated the residual signal after the readout and the associated ripples in the line (Fig. S1b).
- Pairs of crusher gradients (strength = 25mT/m, duration = 1.9 ms) were added around the refocusing pulse, again on all three gradient axes. These crushers were necessary to eliminate the FIDs artefacts resulting from the refocusing pulse. Those artefacts are visible as well in the slice image (see Fig. S2).
- From the pilots reported in Table1, we noted that the 8-channel transmit coil provided more B_1_ and offered acceptable fat suppression with SPIR instead of SPAIR, which allowed the use of shorter TRs. However, very short TRs (< 350 ms) lead to low signal (Fig.S4 of the supplementary material). With both 8-channel and 2-channel transmit coils, SPAIR furnished better fat suppression than SPIR. Different excitation flip angles (FA) were also investigated to optimize the SELINE signal. 146° was found to be the best option in terms of B_1_ homogeneity and relative SNR*,* similar to what was suggested by our simulation in Fig. S3, evaluated according to Diiokio et al. [36], having the maximum signal intensity for a FA of 142°. We evaluated the optimal excitation flip angle for a given set of TR and T1 by minimizing the following equation for the spin-echo transverse magnetization:
  3 $$M_{{xy}} = \frac{{\sin \alpha \left[ {1 - \left( {\cos \beta } \right)e^{{ - TR/T1}} - \left( {1 - \cos \beta } \right)e^{{ - \left( {TR - \frac{{TE}}{2}} \right)/T1}} } \right]}}{{\left[ {1 - \cos \alpha \left( {\cos \beta } \right)e^{{ - TR/T1}} } \right]}},$$
  With $\alpha$ being the varied excitation angle, TR = 500 ms, TE = 50 ms, T1 = 2100 ms (for gray matter at 7 T), β = 180° (FA of the refocusing pulse).
  Overall, a 2-channel transmit acquisition with TR = 500 ms, TE = 50 ms, fat suppression using SPAIR and FA of 146° proved to be the best option, together with the corresponding version with 8-channel transmit. Those sequences were used, respectively for the 5 subjects acquisition on the 2-channel transmit, and 1-subject acquisition on 8-channel transmit with additional runs.

## Results

**Fig. 2:**
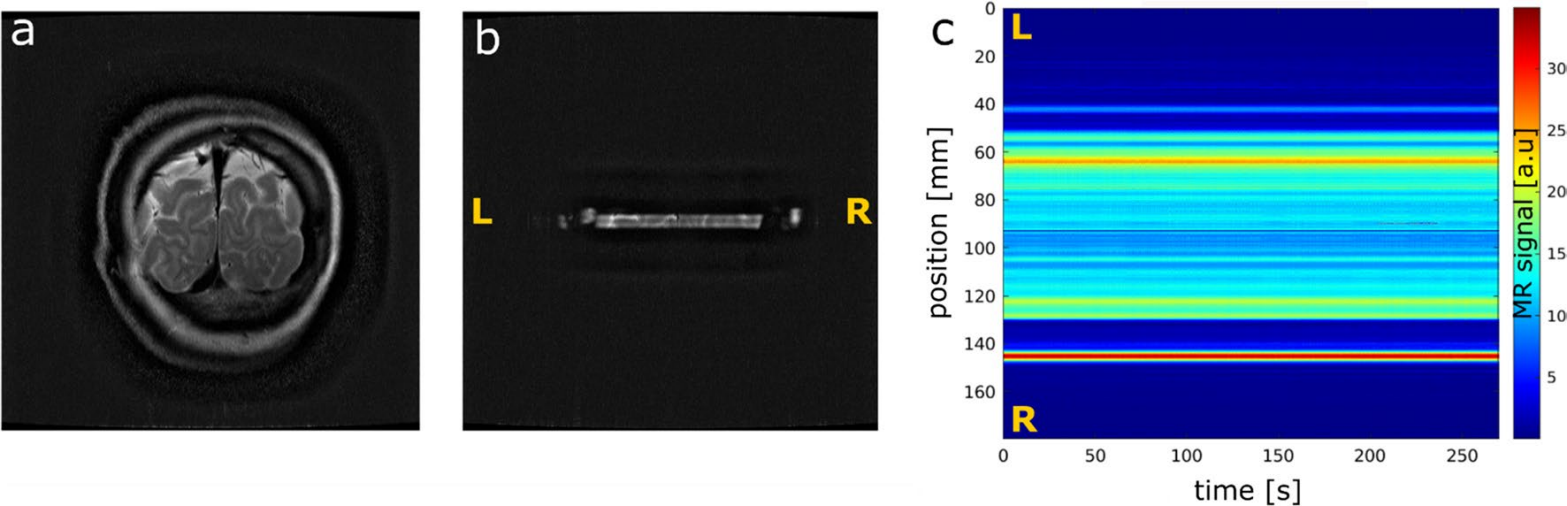
**a** Acquired slice with spin-echo sequence. **b** LSD image for SELINE acquisition, resulting from the intersection of the excited and refocused planes. L and R indicate the left and right direction, respectively. **c** SELINE acquisition, a plot of the MR signal for position and time

Figure 2 shows an example coronal slice (a), the associated LSD image (b), with the signal coming from the intersection of excited and refocused planes, and an example line-scanning acquisition, depicting the evolution of the MR signal for each voxel (position), across time (c).

**Fig. 3:**
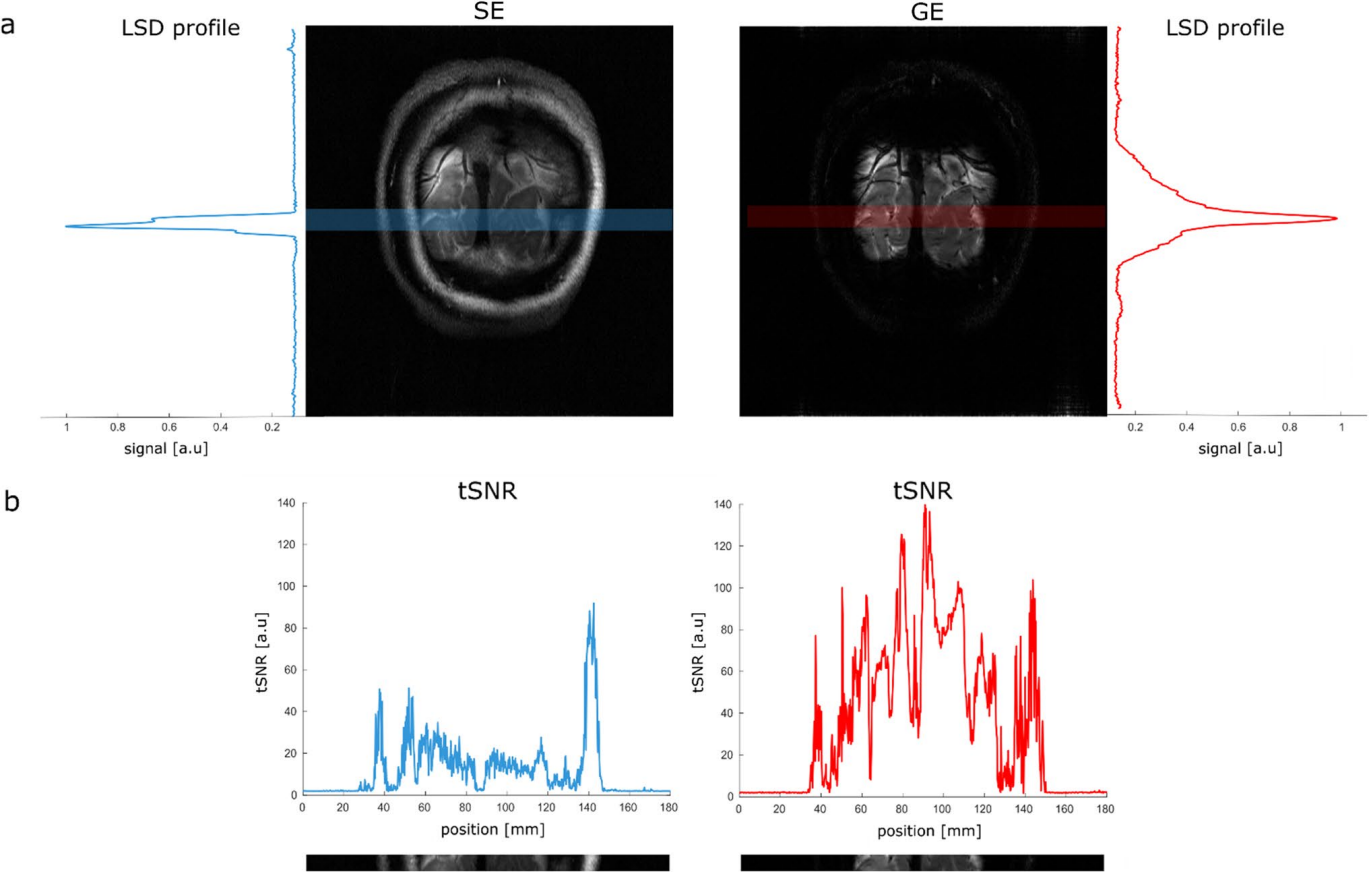
**a** Normalized LSD profile for spin-echo (blue line) and gradient-echo (red line), plotted perpendicularly to the slice, where the region of the line is highlighted in the blue and red box. Note the improved line definition in SELINE. **b** tSNR for spin-echo line-scanning (blue line) and gradient-echo line-scanning (red line), with the anatomical references at the bottom (spatially matched)

In Fig. 3a, a representative participant’s LSD profile is shown for the SE and GE acquisitions, obtained by averaging over all the voxels in the readout direction of an LSD image. Note the much sharper profile of the SE acquisition compared to the GE version, where the effect of imperfect OVS pulses is clearly visible from the residual signal coming from outside the line. On average, across subjects, we found a full-width-at-half maximum (FWHM) of the LSD profile of (5.5 ± 1.0) mm for SELINE and (9.2 ± 3.2) mm for GELINE.

Figure 3b shows the tSNR for line acquisitions with SELINE and GELINE for the same subject. SELINE tSNR values were consistently lower than GELINE tSNR, likely driven by the TE difference as well as the differences in the line profile. In this plot, we evaluated the tSNR after averaging 2 runs of GELINE and 4 runs of SELINE. Across subjects, we evaluated that tSNR was 2.4 times higher for 1 run of GELINE, compared to 1 run of SELINE, and 4 times higher in the 11 voxels ROI containing gray matter.

Results for all 5 participants scanned with the 2-channel transmit system can be found in Fig. S5 showing LSD profiles, tSNR values and timecourses for both GE and SE.

**Fig. 4:**
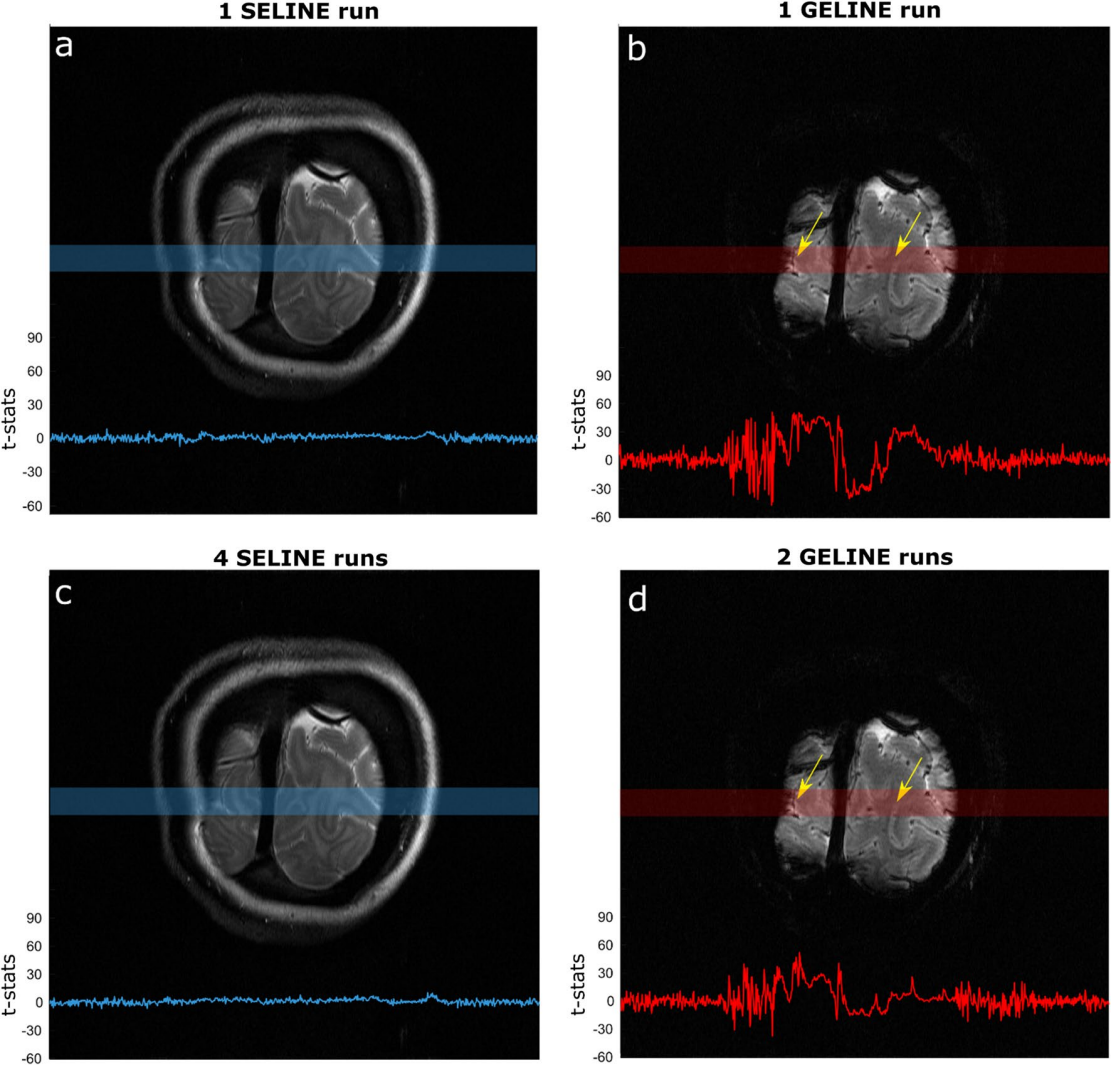
**a** t-stats for 1 SELINE acquisition, **b** 1 GELINE acquisition, **c** 4 runs average SELINE acquisition and **d** 2 runs average GELINE acquisition, overlaid on the acquired slices, for the same representative participant. The light blue and red boxes indicate where the line was positioned, and the yellow arrows highlight gray matter regions

As might be expected from the lower tSNR values, SELINE acquisitions also yielded low functional responses. Figure 4 shows the t-stats values obtained from the GLM of 1 run of SELINE (a) and GELINE (b) and for the average of, respectively, 4 and 2 runs (c and d), overlaid on the acquired slices for SE and GE. While one run of GELINE activation is visible, with relatively high t-stats in the gray matter areas of the line (yellow arrows), even the average of 4 runs of SELINE does not lead to easily detectable responses. Only small responses are visible in the gray matter areas.

On average, across subjects, we found that 1 run of GELINE furnished 3.9 times higher mean value of t-stats along the line, compared to 1 run of SELINE.

**Fig. 5:**
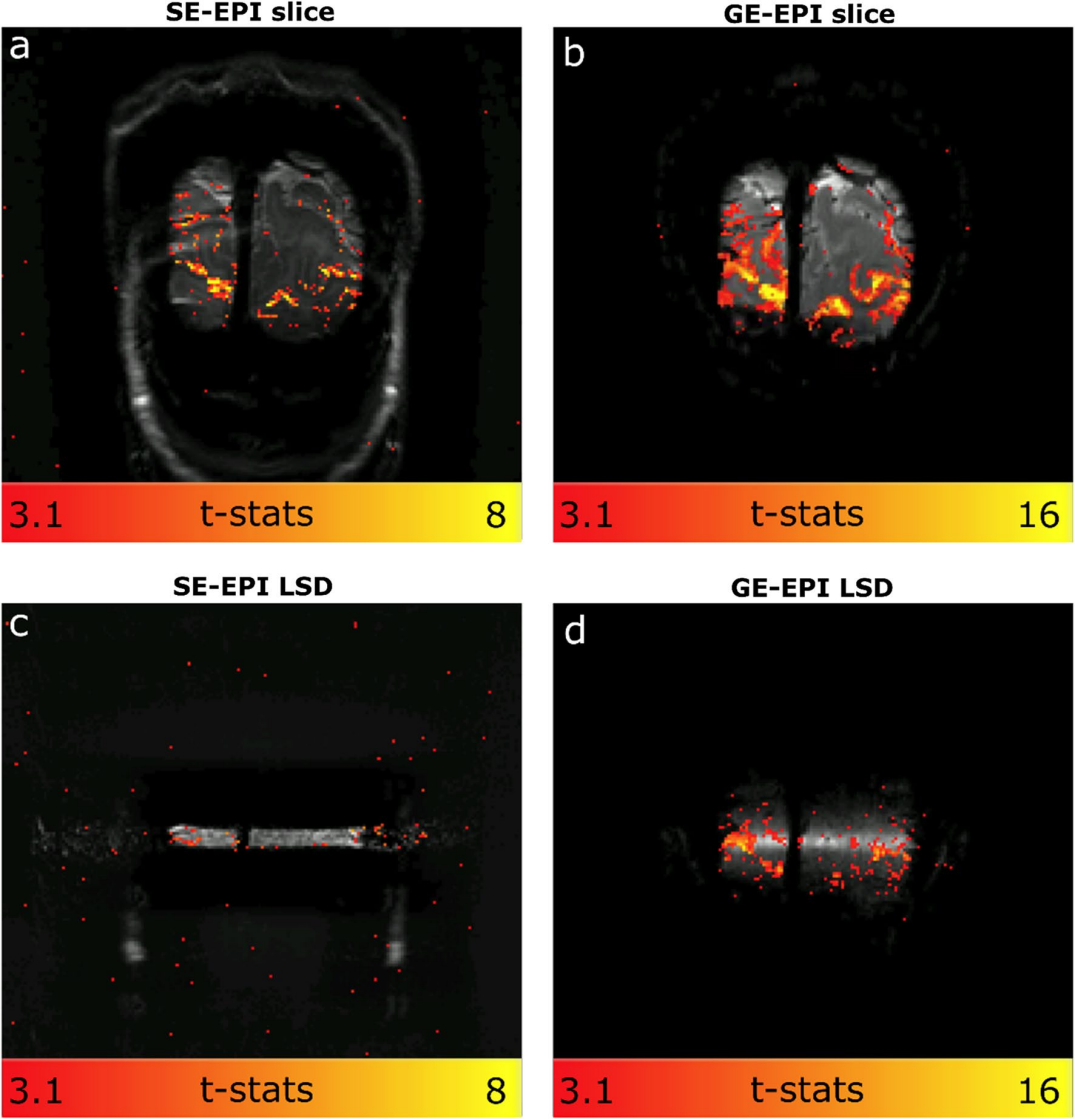
**a** t-stats for SE-EPI slice acquisition, **b** GE-EPI slice acquisition **c** SE LSD image acquisition and **d** GE LSD image. The spatial resolution is 1.5 mm isotropic in all four acquisitions

To investigate the sensitivity differences between the SELINE and GELINE protocols, independent from the line formation, we compared them in a limited-resolution image format as well, for both the slices and LSD images. Figure 5 shows the activation maps of the SE and GE-EPI slices and the accompanying functional LSD images. The SE-EPI showed solid but lower functional responses than GE-EPI. All functional responses were well within the gray matter areas in both SE-EPI and GE-EPI slice acquisitions. Note the different scales for GE-EPI and SE-EPI t-stats. The difference in functional sensitivity between SE-EPI and GE-EPI appeared to be larger in the LSD images (Fig. 5c and d). LSD functional images confirmed good line selection in SELINE but also highlighted the limited available functional signal in SELINE. Note that these voxels were larger than those used in the SELINE and GELINE acquisitions.

**Fig. 6:**
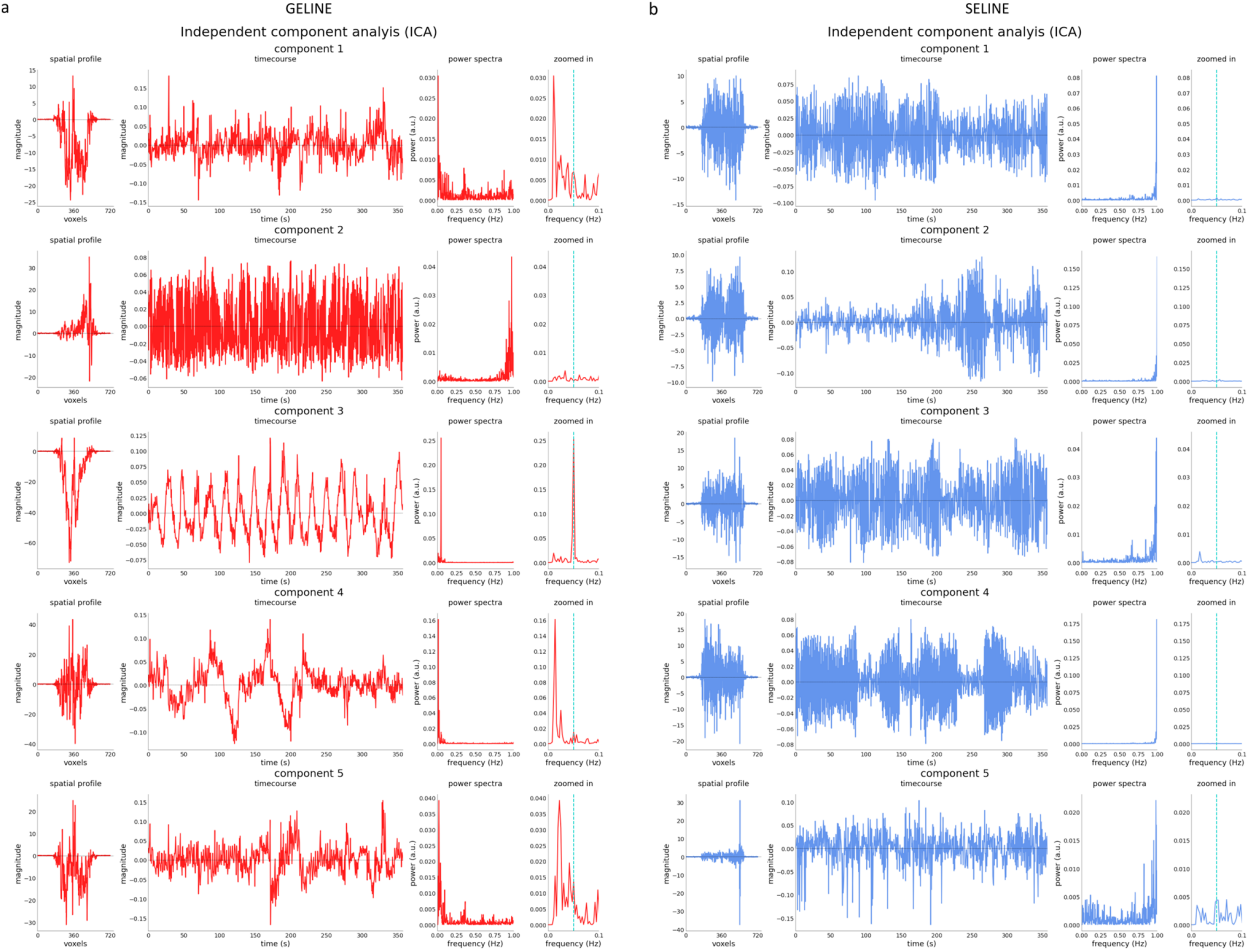
First 5 components of the ICA analysis for GELINE (**a**) and SELINE (**b**) acquisition. For each row we represented the spatial profile, the component timecourse, the power spectra and the power spectra zoomed around the task frequency, indicated by the light-blue dashed bar. Note that the y-axis in the component timecourse is scaled differently for each component

In Fig. 6 we reported the results of the ICA for a representative participant. In GELINE data (a) the task component is observed in one of the ICA’s first components as visible from the time course of the component and the power spectra with the peak at the task frequency (0.05 Hz, indicated by the light-blue dashed bar). For SELINE data the task component was never properly detected by the ICA, indicating that the GLM analysis was not influenced by noisy temporal fluctuations.

## Discussion

In this paper, we reported the first implementation of SELINE for fMRI in humans at 7T. SELINE showed sharp line definition by rotating the refocusing plane relative to the slice acquisition. In SELINE, the line thickness can be easily adapted by simply changing the refocusing gradient strengths.

To achieve SELINE, we used a sinc-shaped refocusing pulse and an optimized gradient crusher and spoiler scheme to remove the signal from spurious echoes. In the vendor implementation of standard SE imaging, no crushers gradients are included, and FID artefacts are located on the edges of the slice; hence they do not interfere with the actual image being acquired. However, when the phase-encoding gradient is removed, to obtain line-scanning data, the artefact signals are concentrated in the line as well. Hence, the crusher gradients were essential for obtaining a clean line signal. Regarding the refocusing pulse shape, the default composite block pulse, used in the standard vendor implementation of SE imaging, resulted in coherence artifacts in the beam. The ‘echo2’ sinc pulse we chose was also used successfully in SE-EPI-based functional acquisitions at 7T [19].

An optimal excitation FA for short TR SELINE was simulated and validated to obtain a maximal SELINE signal. (Fig. S3). We assessed the SELINE fMRI performance by comparing it to GELINE fMRI and image-based comparisons to 2D SE and GE EPI.

We found that within the SE LSD image, it was possible to observe anatomical features of the brain; hence the signal in the line was not affected by the line creation through the rotation of refocusing gradient. On the contrary, the OVS pulses used here for GELINE are positioned in close proximity, and result in some signal deterioration within the line, visible from the decrease of the line-selection fidelity (see also [2]). However, the improved line definition for SELINE leads to a smaller area yielding signal and hence lower tSNR and functional sensitivity. This is in addition to the inherently lower sensitivity of SE-BOLD. The combination of these two effects leads to much lower tSNR and t-stats for SELINE than GELINE.

Regarding the BOLD activation, smaller responses are expected in SE-BOLD weighted data [16]. Much work is undertaken to improve SE-based acquisition for functional imaging [16, 24, 26–28]. Here, we used a single-echo acquisition. We could not detect significant task-driven activation in the SELINE, while GELINE consistently showed clear activation patterns in the visual cortex (on average across participants, t-stats were 3.9 times higher for 1 run of GELINE compared to SELINE). In the image-based comparison presented in Fig. 5, SE-EPI images have clearly defined functional responses located within the gray matter, though at lower t-stats values than GE-EPI. Functional activation is barely detectable in the SE-LSD images, while a clear activation is observed in the corresponding GE-EPI LSD functional images. This difference suggests that there is an additional loss in sensitivity in SE-EPI when generating a line rather than exciting and refocusing an entire slice, possible due to the small size of the target line. The data in Fig. 5 is drawn from a single individual, so it can only show a general trend and is not precise enough to measure effect sizes. Taken into account that the SELINE acquisition has an even lower SNR than the SE-EPI LSD due to the smaller voxel volume (1.8 times smaller) and bigger sampling rate (5 times higher), the SELINE acquisition is currently not suitable for fMRI visual experiments.

Note that we do not expect that inflow effects are more prominent in SELINE compared to GELINE. In fact, in GE sequences, inflow effects arise when the blood within an imaged slice is replaced during the TR, hence, here, in 500 ms. In the case of SE sequences, the critical time during which unwanted inflow effects can occur is shorter because it corresponds to the time between the excitation and the refocusing pulse (TE/2), which is, here, only 25 ms. For fast-flowing blood, the blood will not experience the refocusing pulse and will result in a blood signal reduction effect (wash-out); this is the opposite for GE where one will observe a blood signal increase (inflow effect). For this reason, we may expect (if any) inflow artefacts for GELINE rather than for SELINE.

NORDIC denoising could have an effect in removing task-driven signal from the SELINE data. We also tested unfiltered data and we could not detect any responses due to task either, as visible from Fig.S6 in the Supplementary material.

Another concern that line-scanning often raises is motion. In general, when using line-scanning, motion can be problematic. Although the multi-run data was acquired in a highly experienced individual, some motion is to be expected over the course of 45-min runs. However, since GELINE and SELINE data were acquired in the same session and the same subjects, we would expect any motion problems to be similar in both acquisitions and hence conclude that motion is not the main cause for the observed differences in SELINE and GELINE. Moreover, the current setup does not allow for motion correction, but a prospective motion correction module could be introduced in future implementations [34].

Another theoretically easy source for improvement would be a change in the geometry of the excited and refocused plane when creating the line. In fact, we decided to excite coronally and refocuse axially, not only to match the GELINE and SELINE acquisitions but also due to experimental limitations which we should overcome to be able to perform SELINE acquisition outside of the visual cortex. Being able to excite an axial slice and refocus coronally would lead to less tissue being affected by the refocusing RF, possibly minimizing the FID artefacts mentioned in the “Design considerations”.

Finally, the SELINE sensitivity can be improved in the future by incorporating a multi-echo readout [37], the use of high-density surface coils [38], as well as using higher field strengths. Massive averaging across runs is a widely employed strategy in neuroscience to improve the SNR and recover activation from specific tasks or low-sensitivity acquisitions [39–44]. For SELINE data, we can speculate that averaging across more than 4 runs would help to improve the sensitivity and recover activation detection, at least with a strong block-design visual task. However, such long acquisition times render the data sensitive to motion, especially so at the line-scanning spatial resolution. Prospective motion correction would allow scanning people for a very long time. However, the current implementation of SELINE does not include prospective motion correction [34]. For this reason, at this stage we cannot provide the reader with an estimate of how long one would need to scan to see activation in SELINE data.

The use of a strongly asymmetric spin-echo optimized for BOLD fMRI might also help to increase the detectability of functional activation [45]. Another approach often used to overcome some of the technical limitations of SE is GRASE [23, 46], which has already been suggested for line-scanning purposes at a lower resolution [47]. Moreover, shorter TRs should be properly investigated to fully exploit the power of line-scanning, which promises, at the same time, high spatial and temporal resolution. So far, we noticed that fat suppression using SPIR allows shorter TRs, however, SPIR only suppresses fat adequately when an 8-channel transmit coil is used.

## Conclusion

In this study, we presented our first attempts to implement spin-echo line-scanning in humans at 7T. We demonstrated a much-improved line definition compared to the corresponding gradient-echo version at the cost of lower tSNR and BOLD sensitivity. Due to the non-detectability of active voxels in the visual cortex after a very strong visual task, we conclude that the implementation of spin-echo line-scanning currently lacks adequate sensitivity for line-scanning fMRI. Still, we argue that spin-echo has a high potential for line-scanning applications due to its innate properties of sharp line selection and the microvascular selective functional contrast*.* We believe that several improvements can be performed to further develop the current implementation, which can be considered a starting point for future development. We propose multiple directions for improvement: regarding the SNR increase, high-density surface coils array and higher field strengths, as well as averaging across more runs and a more suitable geometry for the excitation and refocusing plane could play a relevant role. Moreover, the sequence could be optimized with a multi-echo readout (GRASE), asymmetric spin-echo for increased SNR and reduced B_1_ sensitivity [48, 49], and the addition of prospective motion correction.

## Supplementary Information

Below is the link to the electronic supplementary material.

Supplementary file1 (DOCX 3523 KB)

## Data Availability

The data and code that support the findings of this study are available from the corresponding author, upon reasonable request.
