# Supplementary material for: Towards functional spin-echo BOLD line-scanning in humans at 7T
Source: MAGMA. 2023 Jan 10;36(2):317–27. doi: 10.1007/s10334-022-01059-7 (PMC10140128; doi:10.1007/s10334-022-01059-7)
Supplement: Supplementary file 1 — Supplementary file1 (DOCX 3523 KB) [file 10334_2022_1059_MOESM1_ESM.docx]

**Supplementary material**


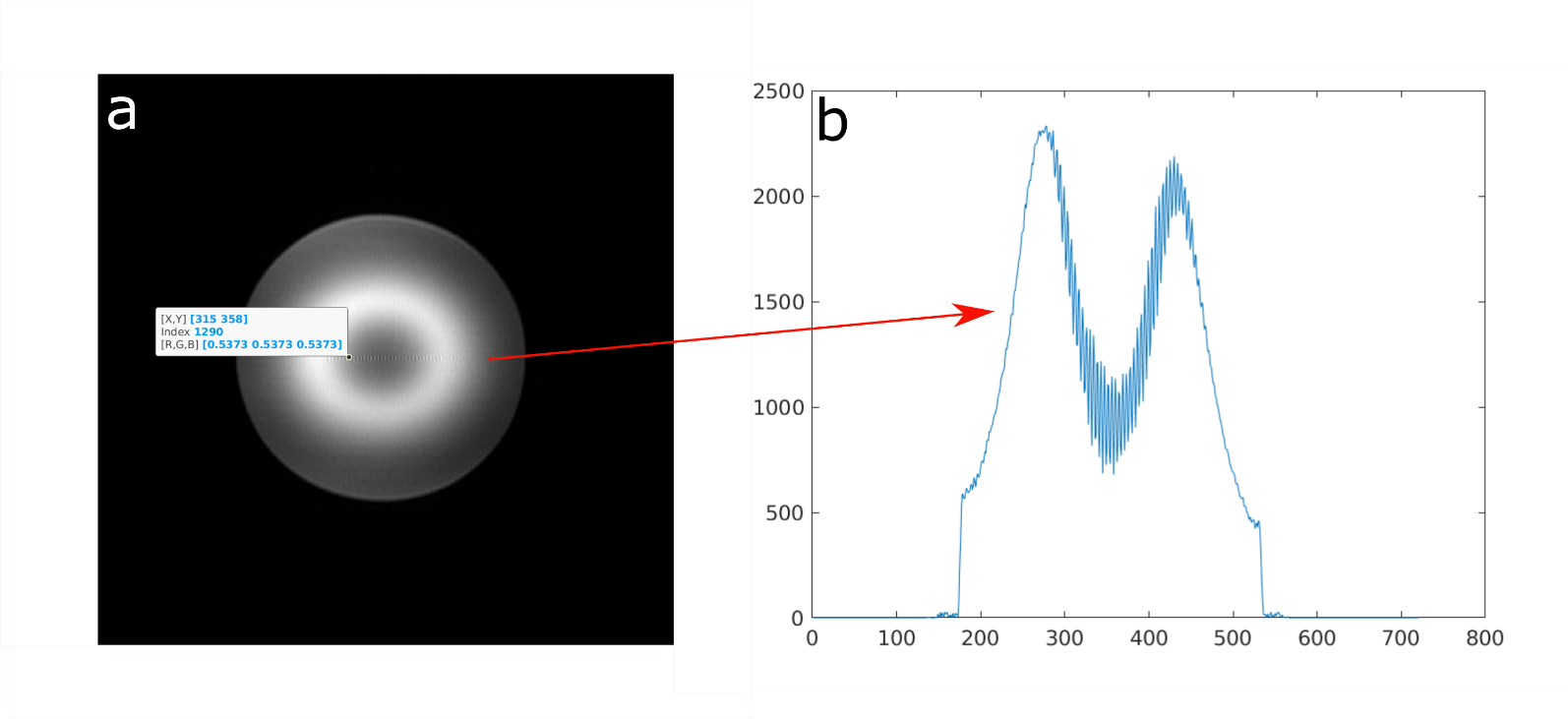


**Fig.S1** Example, on a sphere phantom, of (a) phase artifacts in the slice image and (b) profile of the line artifacts


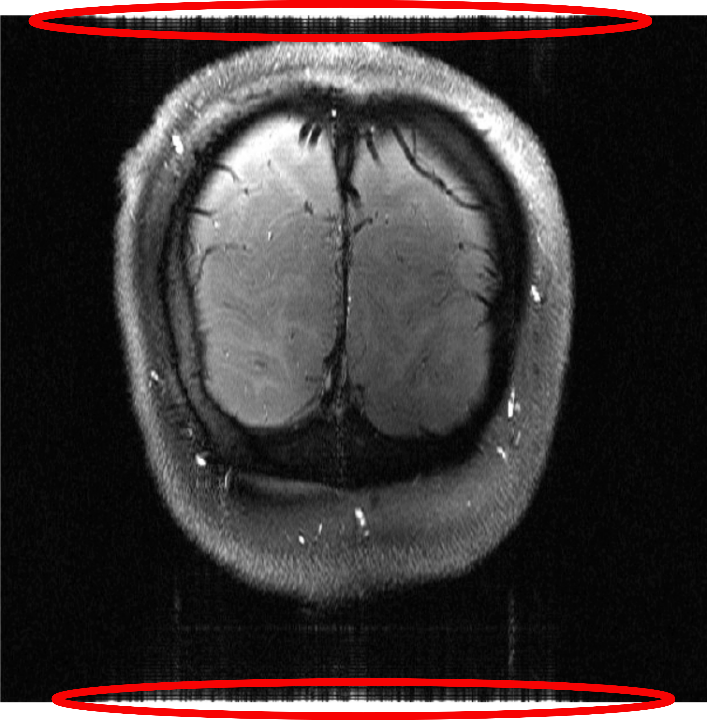


**Fig.S2** Example, on a brain slice, of FID artefacts that would be projected into the line, when the phase encoding gradient is removed. Artefacts are highlighted in the red ellipses


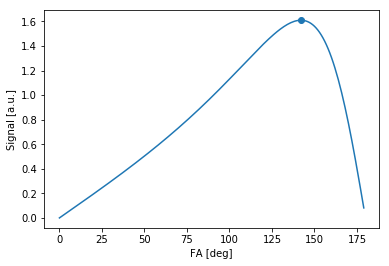


**Fig.S3** Simulation of signal intensity for different flip angles (FA). The maximum signal is obtained for FA = 142°


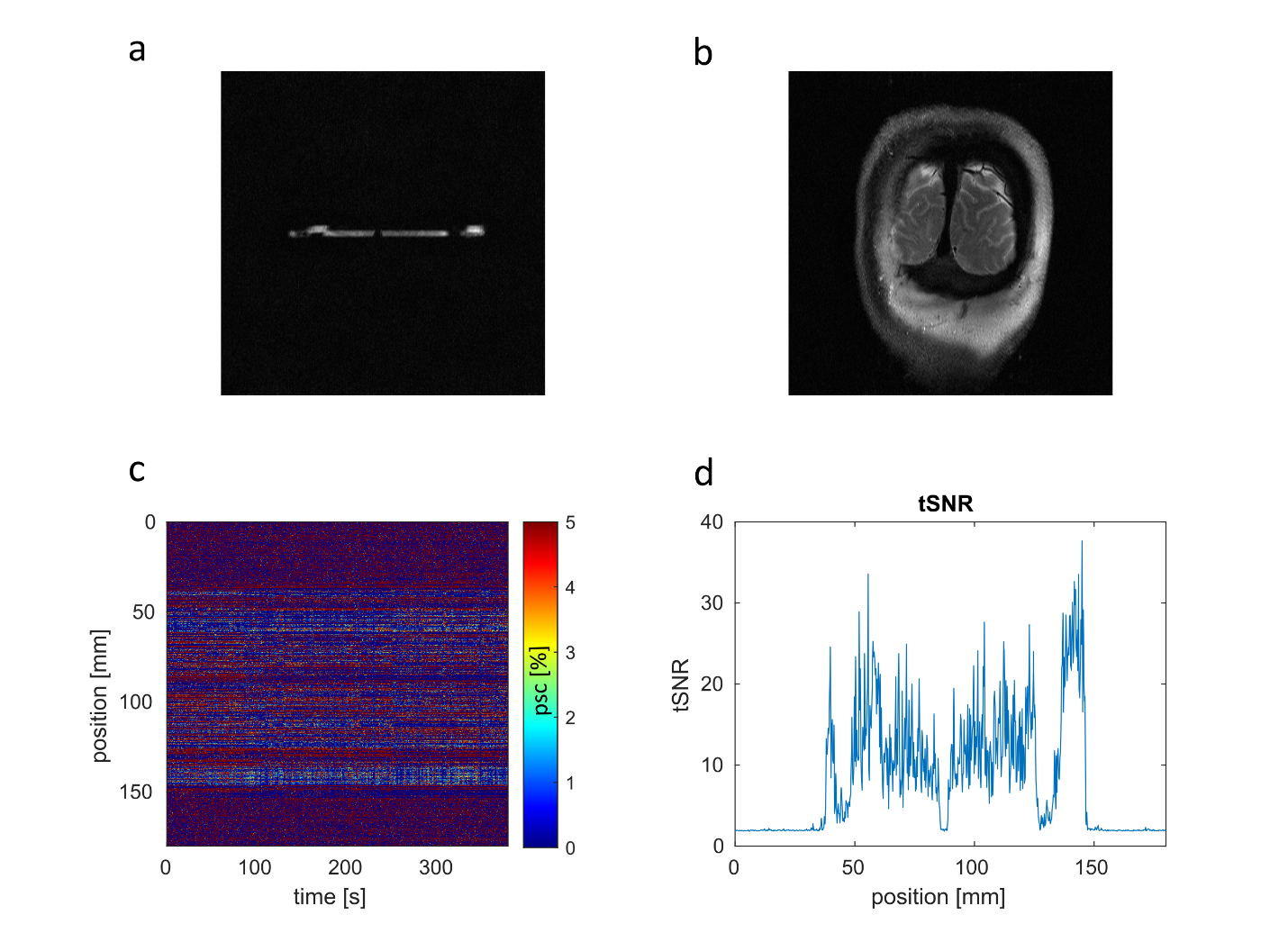


**Fig.S4** (a) LSD image for SE acquisition, (b) slice image, (c) SELINE data in psc and (d) tSNR for the acquisition with TR = 190 ms, acquired on an 8-channel transmit system.


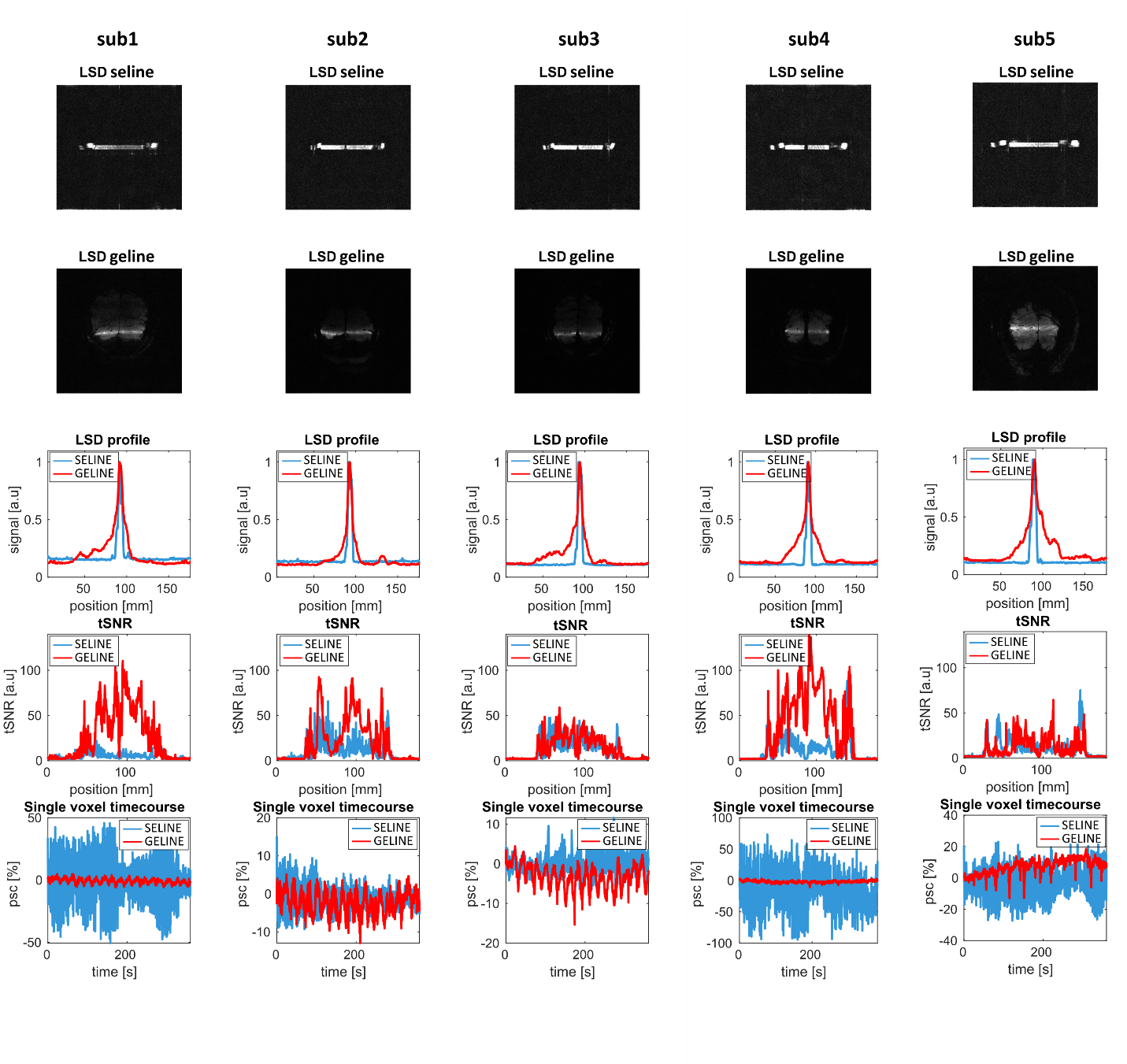


**Fig.S5** Schematic representation of the results for the five subjects scanned with the 2-channel transmit system: LSD images for the SELINE and GELINE acquisition (row 1 and 2 respectively), LSD profiles (row 3), tSNR values along the line (row 4) and single voxel timecourses expressed in psc (row 5), for both GE (red line) and SE (blue line).


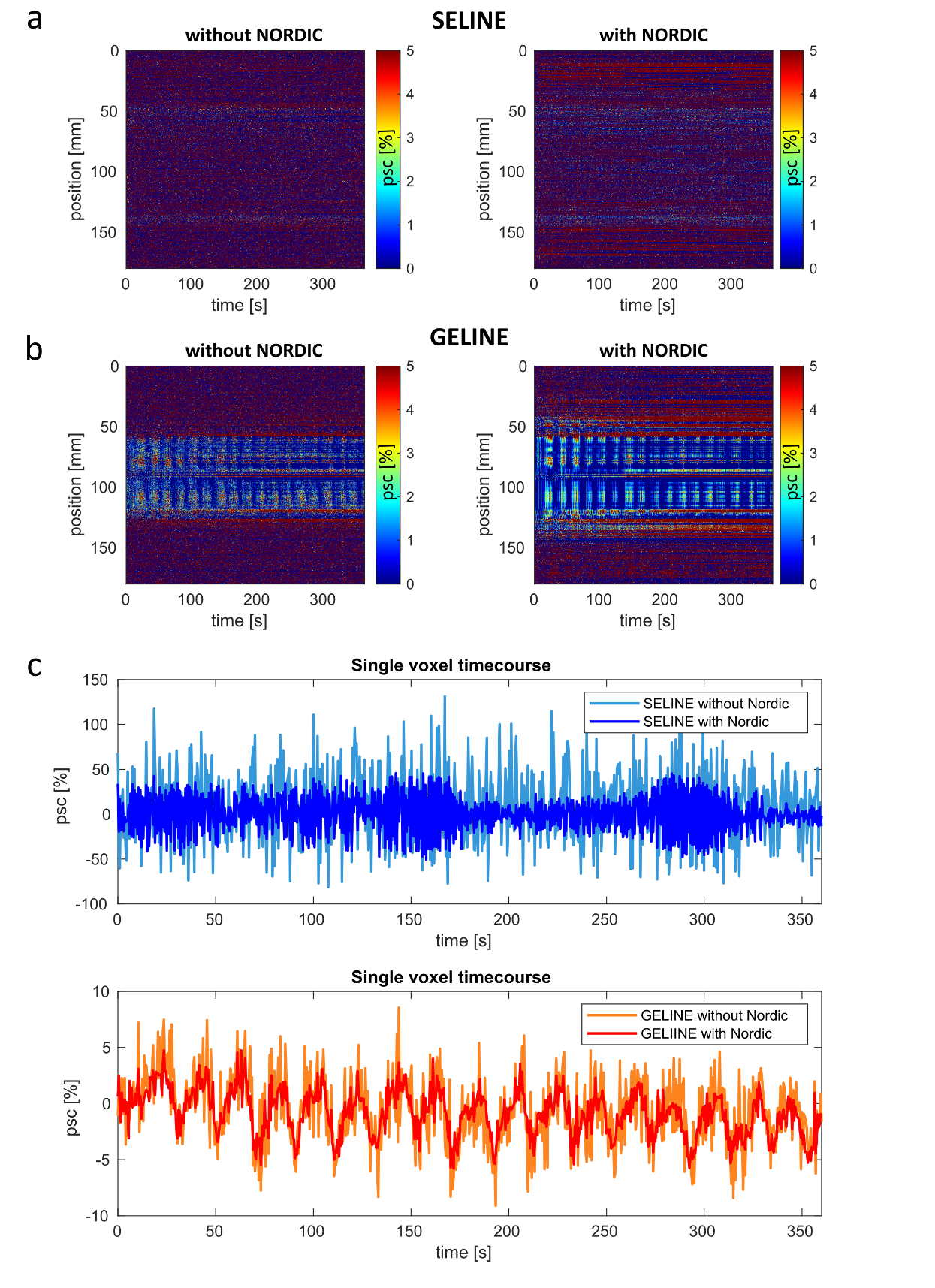


**Fig.S6** Carpet plot in psc for SELINE (a) and GELINE (b) with and without NORDIC-denoising and timecourses in psc with and without NORDIC-denoising for SELINE (c) and GELINE (d), for a representative voxel.
